# Meiotic cohesin Rec8 imposes fitness costs on fission yeast gametes favoring the evolution of parental bias in gene expression

**DOI:** 10.1073/pnas.2524968123

**Published:** 2026-07-02

**Authors:** Celso Martins, Harry Booth, Clàudia Salat-Canela, Zena Hadjivasiliou, Aleksandar Vještica

**Affiliations:** ^a^ Center for Integrative Genomics, Faculty of Biology and Medicine, University of Lausanne, Lausanne CH-1015, Switzerland; ^b^ Mathematical and Physical Biology Laboratory, The Francis Crick Institute, London NW1 1AT, United Kingdom; ^c^ London Centre for Nanotechnology, University College London, London WC1H 0AH, United Kingdom; ^d^ Department of Physics and Astronomy, University College London, London WC1E 6BT, United Kingdom; ^e^ Institute for the Physics of Living Systems, University College London, London WC1E 6BT, United Kingdom

**Keywords:** cell biology, evolution, sexual reproduction, yeast

## Abstract

Differences between partner gametes, which evolved repeatedly in eukaryotes, can contribute to the evolution of the sexes, sexual selection, and non-Mendelian inheritance. Yet, the empirical evidence for how functional asymmetries arise between initially equivalent gametes is limited. Here, we combine theoretical and experimental approaches in the fission yeast *Schizosaccharomyces pombe* to show how selective pressures acting concurrently on gametes and zygotes drive the evolution of gamete differences. We find that despite being morphologically identical, P- and M-type partner gametes invest asymmetrically in zygotic development by contributing different amounts of conserved meiotic cohesins. P-gametes preferentially produce the Rec8 cohesin that increases zygotic fitness but reduces gamete viability, revealing a trade-off between reproductive success and gamete survival. We demonstrate that this asymmetry is mediated by partner-specific communication and model its evolutionary dynamics using empirically determined parameters. Our results support classical theoretical predictions for the evolution of gamete differences and provide a mechanistic understanding of how molecular asymmetries between partners can originate from opposing selection pressures acting in species that lack morphologically distinct gametes.

## Significance

The emergence of distinct male and female gametes is a recurrent transition in the evolution of sexual reproduction, yet how differences between initially equivalent gametes first arise remains poorly understood beyond theoretical models. Using the morphologically indistinguishable gametes of fission yeast, we identify asymmetrically produced factors that improve zygote fitness but simultaneously reduce gamete survival. This creates opposing selective pressures that are optimized when one partner gamete preferentially contributes these resources, favoring the evolution of gamete differences. Our results provide experimental evidence for a mechanism that generates functional asymmetries between partner gametes before morphological differences evolve.

During sexual reproduction, partner gametes fuse to produce a zygote. Zygotic development and fitness are often determined by factors asymmetrically provided by the two parents, and these asymmetries can shape reproductive systems (1–3). In oogamous species, such as animals and flowering plants, large oocytes provision most of the zygote resources, whereas small and numerous sperm are specialized for motility and genome delivery. In contrast, in isogamous species, zygotes rely on similar contributions from the two morphologically identical parents, or isogametes, that differentially express only a handful of mating-type-specific genes (4). How gamete asymmetries arose in isogamous ancestors is a fundamental but open question in biology. The prevailing theoretical models for the evolution of anisogamy—first formalized by Parker, Baker, and Smith (5)—propose that asymmetries between isogametes arise under opposing pressures to 1) increase zygote provisioning, which selects for large, high-investment gametes, and to 2) proliferate gamete numbers, which favors small, low-investment gametes. However, empirical measurements of the proposed selective pressures are largely lacking, as is evidence of molecular mechanisms by which such evolutionary forces can act concomitantly in ancestral isogamous species. In this work, we use fission yeast to show that functional asymmetries operate during sex between isogametes, and we identify molecular basis for the selection pressures that drive the evolution of these asymmetries.

## Results

### Partner Gametes Asymmetrically Produce Meiotic Cohesins.

**Fig. 1.**
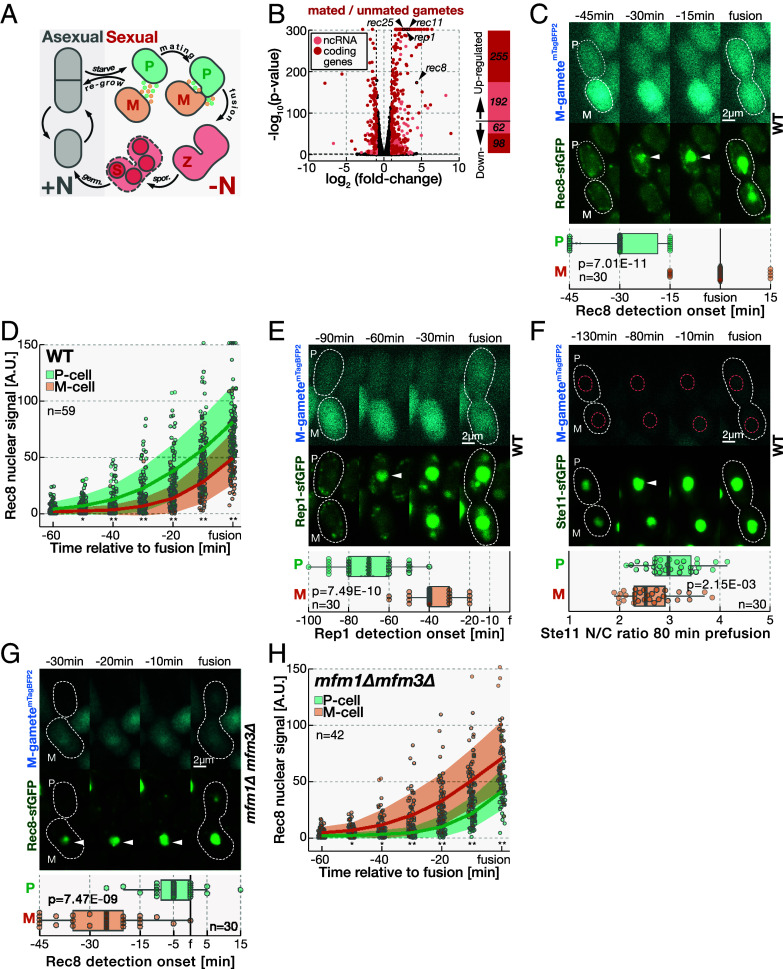
Pheromone-induced signaling drives early expression of meiotic genes in P-gametes. (*A*) Schematic of fission yeast lifecycle. Mitotic proliferation occurs in the presence of nutrients (+N, gray cells) and depletion of a nitrogen source (−N) induces cells to arrest in the G1 cell-cycle phase and to differentiate into partner P- and M-gametes (P and M, respectively). Mating type switching in homothallic h90 strains (13) produces both types of gametes, whereas heterothallic h+ and h− strains produce only P- and M-gametes, respectively (14). Partners communicate using a pair of diffusible pheromones (green and orange dots) and cognate receptors to orient growth that brings them into physical contact. Upon gamete fusion, zygotes (Z) rapidly enter the premeiotic S-phase (15), followed by meiotic divisions that produce four haploid spores (S) within an ascus. When nitrogen source is restored, spores, as well as unfused gametes, resume mitotic cycling (16, 17). (*B*) Volcano plot shows protein-coding (red) and noncoding (pink) polyA+ genes that are differentially expressed [|log2(fold change)| ≥ 1, *P* ≤ 0.05] between nonmated and mated gamete populations 15 h after removal of a nitrogen source (*SI Appendix*, Fig. S1*A*). Black dots indicate genes that are not differentially expressed. Arrowheads indicated meiotic genes upregulated in mating. (*C*) Micrographs show Rec8-sfGFP (green) during homothallic wild-type (WT) cells mating. The M-gamete-specific *p^mam1^* promoter regulates expression of mTagBFP2 (blue), which we used to determine the gametes mating type and time of fusion. Boxplots report the onset of Rec8-sfGFP detection in P- and M-gametes. Note that at −45 min cells show only the faint background fluorescence in the form of dots and strings, most likely corresponding to mitochondria. Note that Rec8-sfGFP produces a diffuse nuclear signal in addition to prominent nuclear foci that are consistent with reported Rec8 enrichment at centromeres (12). Note Rec8-sfGFP expression prior to cell fusion and first in the centrally positioned nucleus of the P-gametes (arrowheads, boxplots), followed by M-gametes, and continued production in zygotes (*SI Appendix*, Fig. S2*A*). We note that Rec8 expression asymmetry between partners was not due to the mTagBFP2 since heterothalic P-gametes carrying the red nuclear marker initiated Rec8-sfGFP production before their unlabeled partners (*SI Appendix*, Fig. S6*B*). (*D*) The graph reports Rec8-sfGFP nuclear signal during WT matings in P- and M-gametes, as indicated. Note that P-gametes induce Rec8 earlier and to higher levels than M-gametes. (*E*) Micrographs show Rep1-sfGFP (green) during WT matings. The mTagBFP2 (blue) as in *C*. Boxplots report the onset of Rec8-sfGFP signal detection in P- and M-gametes. Note that Rep1-sfGFP produces a diffuse nuclear signal which appears first in P-gametes (arrowheads) and subsequently in M-gametes, with continued presence in zygotes (*SI Appendix*, Fig. S2*I*). (*F*) Micrographs show Ste11-sfGFP (green) during WT matings. The mTagBFP2 (blue) as in *C*. Nuclear boundaries, which we visualized with the nucleus-targeted ^NLS^mCherry fluorophore (Movie S1), are indicated with orange dashed lines. Boxplots report the Ste11-sfGFP nucleocytoplasmic (N/C) ratio in P- and M-gametes 80 min before cell fusion. Note that Ste11-sfGFP signal increases during mating and that the P-gamete shows higher nuclear Ste11 levels than the M-gamete at 80 min before fusion (arrowhead, boxplots). (*G*) Micrographs show Rec8-sfGFP (green) during mating of homothallic strains lacking the *mfm1* and *mfm3* genes. The mTagBFP2 (blue) as in *C*. Boxplots report the onset of Rec8-sfGFP signal detection in P- and M-gametes. Note that Rec8-sfGFP signal is produced first in M-gametes (arrowheads, boxplots) during mating of *mfm1∆ mfm3∆* partners. (*H*) The graph reports Rec8-sfGFP nuclear signal during mating between P- and M-gametes lacking *mfm1* and *mfm3* genes. Note that M-gametes induce Rec8 earlier and to higher levels than M-gametes. All timepoints are relative to partner fusion and gamete mating types are indicated (P/M). Scale bars are indicated in micrographs, white dashed lines indicate cell boundaries visualized from brightfield images. Dots represent individual datapoints and we indicate the number of analyzed mating events (n). We show Kruskal–Wallis test *P*-values either as exact values or indicate value range with asterisks as 1.00E-3 > * > 1.00E-6 > **. In boxplots, box edges indicate 25th and 75th percentiles, the central line denotes the median, and whiskers extend 1.5 times the interquartile range. In line graphs, lines connect mean values and shaded areas report SD.

During sexual reproduction, the fission yeast produces morphologically indistinguishable partner P- and M-gametes (Fig. 1*A*), yet the P-parental genome is preferentially utilized to initiate zygotic gene expression (6). Whether gametes differentially invest into zygotic fitness—for example, by depositing zygotic factors before partners fuse—has not been reported. Previous studies suggested that meiotic recombination genes, which have strictly zygotic functions, may become induced already during mating (7–9). To directly test this hypothesis, we compared the transcriptomes of nonmating populations, composed of a single gamete type, with those of mating populations where gametes formed pairs but failed to fuse due to deletion of the formin *fus1* (10, 11) (*SI* *Appendix*, Fig. S1*A*). Mated gametes showed greater than twofold upregulation of 255 protein coding genes (Fig. 1*B*), which included early meiotic genes (7) and genes functionally associated with the meiotic cell cycle and recombination (*SI* *Appendix*, Fig. S1 *B*–*E*). Transcripts for the meiotic cohesin subunits Rec8 (12) and Rec11, and the meiotic recombination factor Rec25, were as abundant as transcripts encoding key mating regulators and housekeeping genes (*SI* *Appendix*, Fig. S1*F*). These results suggest that transcription of meiotic genes may start before gametes fuse.

To characterize the production of meiotic proteins in the two gametes during WT matings, we fused the green fluorescent protein sfGFP (18) with the endogenous Rec8 in homothallic cells, which undergo mating-type switching to produce both P- and M-gametes (Fig. 1*C*). We tracked cytosolic mixing as partners fuse using the blue fluorescent protein mTagBFP2 (19) expressed in M-gametes (Fig. 1*C*). Consistent with transcriptomic analyses, Rec8-sfGFP was produced during mating and before cell fusions (Fig. 1*C* and Movie S1*A*). We detected Rec8-sfGFP also in WT gametes that mated but ultimately failed to fuse with a partner (6.94 ± 1.65% of n > 1,400 mating cells; detailed below). Strikingly, Rec8-sfGFP signal appeared 29 ± 9 min earlier in P- than M-gametes (Fig. 1 *C* and *D*). As partners produced Rec8-sfGFP at comparable rates, the signal in the two gametes remained asymmetric, with M-cells expressing a third of Rec8 of their partners throughout mating (Fig. 1*D* and *SI* *Appendix*, Fig. S2*B*). Tagging endogenous Rec11 or Rec25 with sfGFP revealed similar expression dynamics—both proteins were produced prefusion and first in P-gametes (*SI* *Appendix*, Fig. S2 *C* and *D*). We conclude that key meiotic proteins are produced in a mating-type-specific manner prior to partner fusion and predominantly by P-gametes.

### Pheromone-Driven Partner Communication Generates the Parental Bias in Rec8 Expression.

How do partners bias the Rec8 production? Imaging of nascent transcription sites showed that during mating, *rec8* transcription is initiated first in P-gametes (*SI* *Appendix*, Fig. S2 *E* and *F*). Rec8 expression in gametes was not regulated by the P-cell-specific transcription factor Pi (6) *SI* *Appendix*, Fig. S2*G*). Instead, Rec8-sfGFP induction dynamics (*SI* *Appendix*, Fig. S2*B*) suggested that both gametes regulate Rec8 expression with the same transcriptional machinery that is first activated in P-cells. Consistent with this hypothesis, deleting the Rep1 transcription factor (20, 21) severely reduced Rec8-sfGFP levels in both gametes (*SI* *Appendix*, Fig. S2*H*). Importantly, the endogenous Rep1 tagged with sfGFP appeared in P-gametes 32±16 min earlier than M-gametes (Fig. 1*E* and *SI* *Appendix*, Fig. S2*I* and Movie S1*B*). Rep1 preceded Rec8 production by comparable periods of time in both parents (41 ± 19 and 37 ± 14 min in P- and M-gametes, respectively; *P* = 0.11). The *rep1* promoter activation, and the *rep1* transcription, were also asymmetric and occurred more robustly in P-cells (*SI* *Appendix*, Fig. S2 *J*–*M*). Since these results suggest that the parental bias in Rec8 expression is caused by an earlier asymmetry in *rep1* transcription, we proceeded to investigate *rep1* regulation. We monitored the dynamics of Ste11 HMG family transcription factor that induces Rep1 and numerous mating genes (8, 9, 16, 22). Expectedly (23), in unmated gametes, the sfGFP-tagged endogenous Ste11 localized to the cytosol and the nucleus (Fig. 1*F* and Movie S1*C*). As gametes engaged partners, Ste11-sfGFP nuclear targeting was significantly higher for P- than M-gametes in the period preceding the Rep1 production (Fig. 1*F* and *SI* *Appendix*, Fig. S2*N*). Thus, Ste11 localization, which correlates with its ability to activate transcription of target genes (23), is differentially regulated in partner gametes, and likely responsible for the parental bias in Rep1 and Rec8 expression.

To investigate Ste11 regulation, we tested the roles of diffusible pheromones that control its localization and activity (8, 9, 16, 23). Since P-gametes perceive the partner’s pheromone (16), we deleted *mfm1* and *mfm3* paralogs of the three M-pheromone genes, which did not prevent sexual reproduction (*SI* *Appendix*, Fig. S3*A*). Remarkably, in *mfm1∆ mfm3∆* mutant pairs, Ste11-sfGFP nuclear targeting was stronger in M- than P-gametes (*SI* *Appendix*, Fig. S3 *B* and *C* and Movie S2*A*) and Rep1-sfGFP appeared in M-gametes 37 ± 15 min before P-gametes, effectively reversing the asymmetry observed in WT pairs (*SI* *Appendix*, Fig. S3*D* and Movie S2*B*). Furthermore, *mfm1∆ mfm3∆* mutant M-cells induced Rec8-sfGFP 22 ± 15 min ahead of P-cells (Fig. 1 *G* and *H* and *SI* *Appendix*, Fig. S3*E* and Movie S2*C*). We conclude that distinct properties of pheromone signaling in P- and M-gametes lead to parental bias in the dynamics of Ste11, and expression of Rep1 and Rec8. Collectively, our results support a model where differences in pheromone signaling between gametes lead to distinct Ste11 nuclear targeting and activity that generate parental bias in the expression of Rep1 and its target genes, including meiotic segregation and recombination factors Rec8, Rec11, and Rec25.

### Rec8 Produced in Gametes Promotes Zygotic Success and Offspring Survival.

**Fig. 2.**
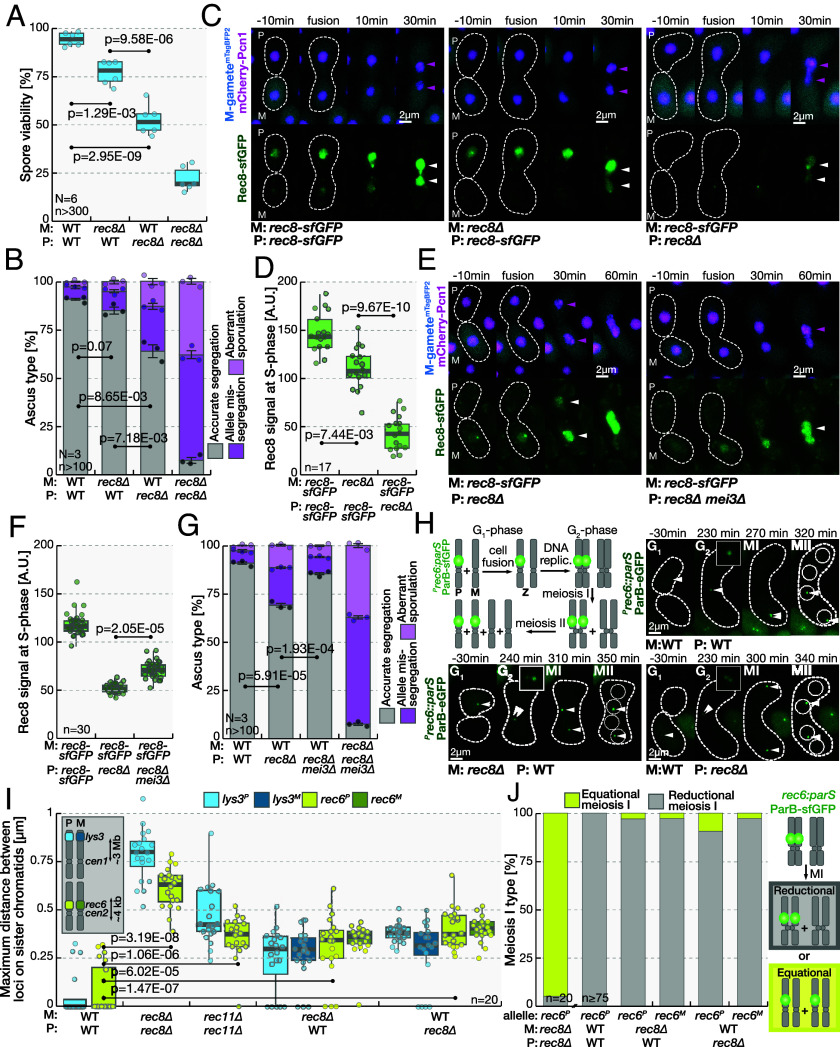
The early induction of Rec8 in P-gametes is required for successful meiosis. (*A*) Boxplots report the viability of spores produced in indicated crosses of WT and *rec8∆* mutant gametes. Tukey test *P*-values are shown. Note that deleting *rec8* in the P-gamete reduces spore viability more than deleting *rec8* in the M-gamete. (*B*) Stacked histograms report the frequency of asci with accurate meiotic segregation of reporter alleles (gray; *SI* *Appendix*, Fig. S4*D*), asci with mis-segregate alleles (purple) and asci containing aberrant spore number (pink) for indicated crosses between WT and *rec8∆* mutant gametes. Welch test *P*-values are shown. Note that meiotic defects are more pronounced when *rec8* is deleted in P- than M-gametes only. (*C*) Micrographs show Rec8-sfGFP (green) expressed from either one or both parental alleles, as indicated. Cells coexpress mCherry-Pcn1 (magenta), which during DNA-replication forms foci (magenta arrowheads) and a uniform nuclear signal outside S-phase. The M-gamete-specific *p^mam1^* promoter regulates expression of mTagBFP2 (blue), which we used to determine the mating type of gametes and the time of fusion. Note that the Rec8-sfGFP signal at the onset of S-phase (white arrowheads) is lower when produced by the M-allele alone (*Right*) than that produced by the P-allele alone (*Middle*) or both parental alleles (*Left*). (*D*) The boxplot reports Rec8-sfGFP levels at the onset of S-phase in zygotes produced in indicated crosses shown in *C*. Dunn test *P*-values are shown. Note that Rec8 levels at S-phase are largely produced by the P-allele. (*E*) Micrographs show Rec8-sfGFP (green) expressed from the M-allele in indicated crosses where P-gametes lack the *rec8* gene and the *mei3* gene is either intact or deleted. The S-phase marker mCherry-Pcn1 and mTagBFP2 as in *C*. Note that at the premeiotic S-phase onset (magenta arrowheads) is delayed when P-gametes lack the *mei3* gene and that this allows for increased Rec8-sfGFP production from the M-allele (white arrowheads). (*F*) The boxplot reports Rec8-sfGFP levels at the onset of S-phase in zygotes produced in indicated crosses shown in (*E*). Dunn test *P*-values are shown. Note that deleting the *mei3* in P-gametes, which delays premeiotic S-phase, allows for higher Rec8 production from the M-allele by the time S-phase starts. (*G*) As in *B*, stacked histograms report meiotic segregation accuracy for indicted crosses between WT and *rec8∆* mutant gametes where the *mei3* gene in P- gametes is either intact or deleted. Welch test *P*-values are shown. Note that the deletion of the *mei3* P-allele, which delays premeiotic S-phase, largely suppresses meiotic segregation defects caused by the deletion of the *rec8* P-allele. (*H*) The schematic illustrates the WT meiotic dynamics of chromosomes (gray) inherited by the zygote (Z) from P- and M-parents, where the *rec6* P-allele located ~4 kb from the centromere (gray circle) is labeled (green). Micrographs show mating between indicated WT, *rec8∆,* and *rec11∆* partners where P-gametes carry the *rec6* locus labeled with the *parS^c3^*/ParB^c3^-eGFP (green) and M-gametes carry the *rec6* locus labeled with the *parS^c2^*/ParB^c2^-mCherry-NLS (Movie S8). Cell cycle phases are indicated (G_1_, G_2_, MI, and post-MII). Note that the *rec6* locus-recruited *parS^c3^*/ParB^c3^-eGFP (arrowheads, insets) produces a single dot prior to meiosis in WT zygotes but two dots in zygotes that lack either parental *rec8* allele. Also note that at meiosis I, in WT zygotes the *rec6* loci cosegregate and in *rec8* mutants the foci separate. (*I*) As indicated, boxplots report the maximum separation between genomic loci (*lys3* or *rec6*, *Inset*) residing on sister chromatids of indicated parental chromosome (P or M) prior to meiosis in zygotes produced in indicated crosses of WT, *rec8∆,* and *rec11∆* gametes. The genomic loci were visualized using the *parS^c3^*/ParB^c3^-eGFP reporters shown in *C* and *SI* *Appendix*, Fig. S5*A*. Note that the removal of parental *rec8* alleles reduces loci cohesion in zygotes. (*J*) For indicated crosses between wild-type and *rec8∆* mutant gametes, stacked histograms report the frequency of *rec6* loci residing on sister chromatids of indicated parental chromosome (P or M) cosegregating together (reductional division) or separating (equational division) at meiosis I. The visualized *rec6* loci are shown in panel *H*. Note that the zygotes lacking the *rec8* P-allele show higher rates of aberrant, equational meiosis I than zygotes lacking the M-allele. All timepoints are relative to cell fusion and gamete mating types are indicated (P/M). Scale bars are indicated in micrographs, white dashed lines indicate cell boundaries visualized from brightfield images. Dots represent individual datapoints and we indicate the number of analyzed replicates (N) and relevant cell types (n). In stacked histograms, bars report means and error bars denote SD. In boxplots, box edges indicate 25th and 75th percentiles, the central line denotes the median, and whiskers extend 1.5 times the interquartile range.

Having observed differential Rec8 provisioning by the two parents, we proceeded to test whether *rec8* P- and M-alleles make distinct contributions to zygote development. We measured the viability of spores produced in crosses of *rec8* mutant and WT heterothallic strains, which do not undergo mating-type switching and produce gametes of a single mating type. Strikingly, deleting *rec8* only in the P-gamete reduced progeny viability by 47 ± 8%—nearly twice the 24 ± 7% reduction observed upon deleting *rec8* in M-gametes only (Fig. 2*A*). In contrast, loss of a single *rec8* copy did not reduce the viability of spores produced by azygotic meiosis (24) in diploid strains since heterozygous *rec8∆/rec8^WT^* and homozygous *rec8^WT^/rec8^WT^* diploids produced spores of similar viability (*SI* *Appendix*, Fig. S4*A*). Two lines of evidence show that during sex, *rec8* parental alleles make quantitatively distinct contributions to accuracy of meiosis (12, 25); First, timelapse imaging of the fluorophore tagged Hht1 histone H3 (26) revealed that meiotic divisions failed to segregate chromatin into four even masses in 30 ± 5% of zygotes produced by *rec8*∆ P-parent, compared to 14 ± 6% of zygotes with *rec8∆* M-parent (*SI* *Appendix*, Fig. S4 *B* and *C* and Movie S3). Second, we monitored the inheritance of two parental reporter alleles of the same genomic locus (*SI* *Appendix*, Fig. S4*D*) and found that meiotic allele segregation was perturbed more strongly upon deleting *rec8* in P-gametes compared to M-gametes (23 ± 3% and 10 ± 2% of mis-segregation events, respectively; Fig. 2*B*). Consistent with meiotic defects, the frequency of asci with aberrant spore number was 13 ± 3% and 5 ± 2% for crosses where *rec8* was deleted in either P- or M-gametes, respectively (Fig. 2*B*). Thus, during sex, parent-of-origin effects govern Rec8 contributions to allele segregation and spore viability, with the P-allele exerting significantly greater impact.

Even though M-pheromone defective mutants (*mfm1∆ mfm3∆*) strongly delayed Rec8 expression in P-gametes (Fig. 1 *G* and *H*), we detected only mild defects in sporulation and meiotic allele segregation (*SI* *Appendix*, Figs. S3*A* and S4*E*). These findings suggested that during sex between *mfm1∆ mfm3∆* mutants, *rec8* P-allele has a limited role. Indeed, *mfm1∆ mfm3∆* mutant zygotes lacking the *rec8* P-allele showed 10 ± 2% fewer meiotic defects than those lacking the M-allele (*SI* *Appendix*, Fig. S4*E*). Thus, in contrast to WT, meiosis in *mfm1∆ mfm3∆* zygotes predominantly relies on *rec8* supplied by M-gametes. This suggested that, in principle, Rec8 provisioned by either gamete supports its functions. Consistent with this idea, expressing Rec8 from the *p^mam1^* promoter, which drives expression of the pheromone receptor in M-gametes early in mating, fully rescued meiotic defects in zygotes that lacked native *rec8* loci (*SI* *Appendix*, Fig. S4 *F* and *G* and Movie S4*A*). The loss of native *rec8* alleles was also rescued when Rec8 was induced from the *p^pak2^* promoter, which drives expression of the Pak2 polarity kinase during mating and in both gametes (*SI* *Appendix*, Fig. S4 *G* and *H* and Movie S4*B*). However, when Rec8 was placed under the *p^mei3^* promoter, which is rapidly and exclusively activated following gamete fusion to express the Mei3 meiotic inducer (15, 27), it failed to efficiently complement the native *rec8* loci and mating produced 30±3% of defective asci (*SI* *Appendix*, Fig. S4 *G* and *I* and Movie S4*C*). These findings suggest that the roles of *rec8* parental alleles are not strictly determined by the parent of origin, and instead, relate to the time of Rec8 induction relative to gamete fusion.

Since gamete fusion is closely followed by premeiotic S-phase (15)—and because sister chromatid cohesion requires cohesin loading onto chromosomes before DNA replication (28–30)—we hypothesized that Rec8 production in gametes ensures sufficient levels at zygotic S-phase. To quantify Rec8 amounts produced from each parental allele at S-phase, we monitored crosses where one or both parents carried *rec8-sfGFP* and covisualized the mCherry-tagged DNA replication fork component Pcn1 (31), which forms nuclear foci only during DNA replication (31) (Fig. 2*C* and Movie S5). Compared to expression from both parental copies, P- and M-alleles produced 75 ± 18% and 29 ± 13% of the Rec8-sfGFP at premeiotic S-phase (Fig. 2*D*). DNA replication was initiated at comparable times postfusion in all crosses (*SI* *Appendix*, Fig. S4*J*). These findings suggested that the *rec8* M-allele cannot compensate for the loss of the P-allele because Rec8 production in M-gametes starts too close to S-phase. To test this idea, we performed crosses where P-gametes lacked the *rec8* gene and we delayed the premeiotic S-phase by 35 ± 13 min by deleting one copy of the zygotic inducer *mei3* (6, 15) (*SI* *Appendix*, Fig. S4*K* and Movie S6). This delay allowed a 38 ± 22% increase in Rec8-sfGFP production from the M-allele at S-phase (Fig. 2 *E* and *F*) and, importantly, largely rescued meiotic defects caused by *rec8* P-allele deletion (Fig. 2*G*). Mutating *mei3* did not suppress defects in control zygotes that lacked both *rec8* alleles (Fig. 2*G*). We conclude that *rec8* induction in P-gametes ensures sufficient Rec8 levels at premeiotic S-phase to support sister chromatid cohesion.

To further understand how reduced Rec8 levels affect meiotic chromosomes, we used the *parS/ParB* CTPase reporter system (32, 33) to visualize the centromere-distal *lys3* and the centromere-proximal *rec6* loci (*Materials and Methods*), which are located ~3 Mb and ~4 kb from the centromeres of chromosomes I and II, respectively (Fig. 2*H* and *SI* *Appendix*, Fig. S5 *A* and *B* and Movies S7 and S8). In WT zygotes, replicated loci residing on sister chromatids appeared almost exclusively as a single dot indicative of tight cohesion (Fig. 2 *H* and *I* and *SI* *Appendix*, Fig. S5*A*). Rec8 from both parents was required for efficient sister chromatid cohesion at both the centromere-distal and centromere-proximal loci in zygotes (Fig. 2 *H* and *I* and *SI* *Appendix*, Fig. S5*A*; ref. 34). Importantly, in zygotes lacking one parental *rec8* allele—and more often in those lacking the P- than the M-allele—we observed that centromere-linked *rec6* loci on sister chromatids segregated to opposite poles (Fig. 2 *H* and *J*), which is indicative of aberrant, equational first meiotic division. This is in contrast to WT zygotes that carried out reductional meiosis I, where *rec6* loci residing on sister chromatids cosegregated together (Fig. 2 *H* and *J*). Interestingly, deletion of the *rec8* P-allele had a more pronounced effect on the chromosome inherited from the P-parent (Fig. 2*J*). The observed equational meiosis I suggested that ablation of one *rec8* parental allele reduces the ability of meiotic centromeres to orchestrate reductional meiosis (34). Even though removal of one *rec8* parental allele reduced cohesion on chromosome arms (Fig. 2*I* and *SI* *Appendix*, Fig. S5*A*; ref. 34, 35), nondisjunction of homologs at meiosis I—where four *rec6* loci on replicated homologues segregate together (34)—occurred very rarely and at rates comparable to WT zygotes (*SI* *Appendix*, Fig. S5 *B* and *C*). However, when we specifically impaired chromosome arm cohesion by deleting the meiotic cohesin Rec11 (34, 35), homologue nondisjunction rates increased and the spore viability was reduced (ref. 34, 36 and *SI Appendix*, Fig. S5 *B*–*D* and Movie S9). Consistent with our observations that P-gametes are first to induce Rec11 (*SI* *Appendix*, Fig. S2*C*), we find that the *rec11* P-allele contributed more to spore viability than the M-allele (*SI* *Appendix*, Fig. S5 *D* and *E*). Deleting both *rec11* and *rec8* in P-gametes did not show additive effects (*SI* *Appendix*, Fig. S5*E*; ref. 36). These results suggest that meiotic cohesins produced in P-gametes promote spore viability primarily through Rec8 functions at centromeres with a smaller role for Rec11 and cohesion at chromosome arms.

Collectively, our results are consistent with a model that by producing distinct amounts of meiotic cohesins ahead of S-phase, P- and M- parental alleles differentially contribute to the meiotic chromosome cohesion and centromere function needed for efficient meiotic divisions and production of viable spores.

### Rec8 Causes Genomic Instabilities and Reduced Viability in Gametes.

**Fig. 3.**
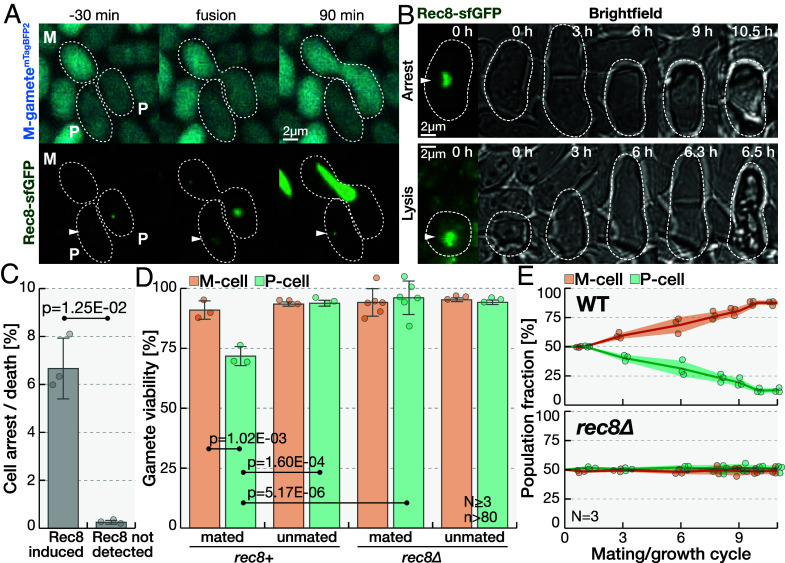
Rec8 accumulated during mating increases genomic instabilities and reduces fitness of P-gametes. (*A*) Micrographs show Rec8-sfGFP (green) in homothallic mating mixtures where two P-gametes compete for one M-partner. The M-gamete-specific *p^mam1^* promoter regulates expression of mTagBFP2 (blue), which we used to determine the gametes mating type and time of fusion. Indicated times are relative to cell fusion. Note that Rec8-sfGFP is expressed prefusion in both outlined P-gametes (arrowheads) and that one of them fails to fuse with a partner. (*B*) The timelapses of brightfield images (gray) show homothallic cells returned to growth media after being induced to mate. Rec8-sfGFP (green) is shown at the timelapse onset. Note that for gametes that induce Rec8, mitosis leads to cell lysis (*Bottom*) or the daughter cells that arrest growth (*Top*). (*C*) The bar chart reports the frequency of growth arrest and cell death for mitotic progeny of gametes that either induced or did not show detectable Rec8 signal at the time homothallic mating mixtures were placed on growth media. Welch test *P*-values are shown. Note that the gametes with notable Rec8 expression have a ~10-fold increase in cell death. (*D*) The bar chart reports viability (*SI* *Appendix*, Fig. S6*D*) of mated and unmated P- and M-gametes with indicated genotypes. Tukey test *P*-values are shown. Note that mating reduces viability of P-gametes and that this is dependent on the *rec8* gene. (*E*) Graphs report the fraction of P- and M-gametes after the indicated number of experimental cycles of mating and regrowth (*SI* *Appendix*, Fig. S6*H*) for evolving populations of heterothallic *fus1∆* cells that either carry WT *rec8* (*Top*) or lack the *rec8* gene (*Bottom*). Note that M-gametes outcompete P-gametes in populations with WT *rec8* allele but not in populations where *rec8* is deleted. Scale bars are indicated in micrographs, white dashed lines indicate cell boundaries visualized from brightfield images and gamete mating types are indicated (P/M). Dots represent individual datapoints and we indicate the number of analyzed replicates (N) and relevant cell types (n). In bar plots, bars report means and error bars denote SD. In line graphs, lines connect means, and shaded areas report SD.

While preceding results show that Rec8 production in gametes promotes zygotic fitness, we find that it comes at a fitness cost to gametes. In mating populations of Rec8-sfGFP homothallic strain, we observed gametes that induced Rec8 during mating but then failed to fuse with a partner. These gametes typically arose when two P-gametes competed for a mate and only one fused with a partner, leaving the unfused P-gamete with induced Rec8 (6.33 ± 1.38% of mating events; Fig. 3*A* and Movie S10) Additionally, when we interrupted mating by transferring mating mixtures to growth media, Rec8-expressing unfused gametes constituted 7.1 ± 3.4% of the population. These gametes were predominantly P-cells, as indicated by their lower expression from the M-gamete-specific promoter compared to Rec8-negative gametes (*SI* *Appendix*, Fig. S6*A*), a conclusion we confirmed using labeled heterothallic partners (*SI* *Appendix*, Fig. S6 *B* and *C*). Thus, WT mating mixtures contain a subpopulation of Rec8-expressing haploid gametes that fail to fuse and are predominantly P-mating type. Importantly, when the supplied nutrients triggered mitotic reentry, 6.7 ± 1.3% of Rec8-expressing gametes—or their daughters produced in first division—either lysed or arrested growth (Fig. 3 *B* and *C* and Movie S11). In contrast, such defects arose in only 0.3 ± 0.1% of gametes that did not induce Rec8 (Fig. 3*C* and Movie S11). These results suggested that Rec8 expressed during mating compromises cell viability, which may explain why Rec8 is supplied in a mating-type-specific manner despite its crucial role for meiosis.

The correlation between Rec8 induction and cell viability (Fig. 3*C* and ref. 35), prompted us to directly test the role of Rec8 in survival of unfused gametes. We prepared mating mixtures of *rec8+* and *rec8∆* cells—where we deleted the *fus1* gene to abolish cell fusion—and we measured the viability of unmated gametes and gametes that formed mating pairs (*SI* *Appendix*, Fig. S6*D*). Remarkably, mating reduced viability of P-gametes by 22 ± 4%, and this viability loss was dependent on the intact *rec8* gene (Fig. 3*D*). Deleting the *rec11* gene also increased viability of mated P-gametes albeit with a smaller effect (*SI* *Appendix*, Fig. S6*E*), suggesting that recruitment of the meiotic cohesin complex to both centromeres and chromosome arms reduces gametes viability. In contrast, mating and *rec8* or *rec11* genes did not strongly impact the viability of M-gametes (Fig. 3*D* and *SI Appendix*, Fig. S6*E*). The loss-of-heterozygosity assay (35) (*SI* *Appendix*, Fig. S6*F*), which reports mitotic mis-segregation of alleles, showed a Rec8-dependent, 17 ± fivefold increase in mitotic defects for mated P-cells as compared to unmated cells with the same genotype (*SI* *Appendix*, Fig. S6*G*). Taken together, our results show that Rec8 accumulated during mating of P-gametes that fail to fuse with a partner increases genomic instabilities and reduces their fitness.

Our results suggest that the asymmetry in *rec8* expression causes distinct fitness costs on P- and M-gametes and, thus, may affect how gamete populations evolve. To test this idea, we mated equal number of *fus1∆* heterothallic P- and M-gametes, regrew the mated population and used this population to repeat the cycle (*SI* *Appendix*, Fig. S6*H*). Strikingly, the fraction of P-gametes in the population decreased to 41 ± 3% after only three experimental cycles and continued to decrease in subsequent cycles reaching 13 ± 2% of the recovered cells after eleven mating rounds (Fig. 3*E*). In parallel, we subjected *rec8∆ fus1∆* populations of P- and M-gametes to the same cycles of mating and regrowth and found no evidence for selection of either gamete type (Fig. 3*E*). We conclude that *rec8* differentially affects the fitness of the two gamete types during sexual and asexual reproduction, which may contribute to the evolution of asymmetric functions in gametes.

### Rec8-Dependent Costs and Benefits Drive the Evolution of Parentally Biased Rec8 Expression.

**Fig. 4.**
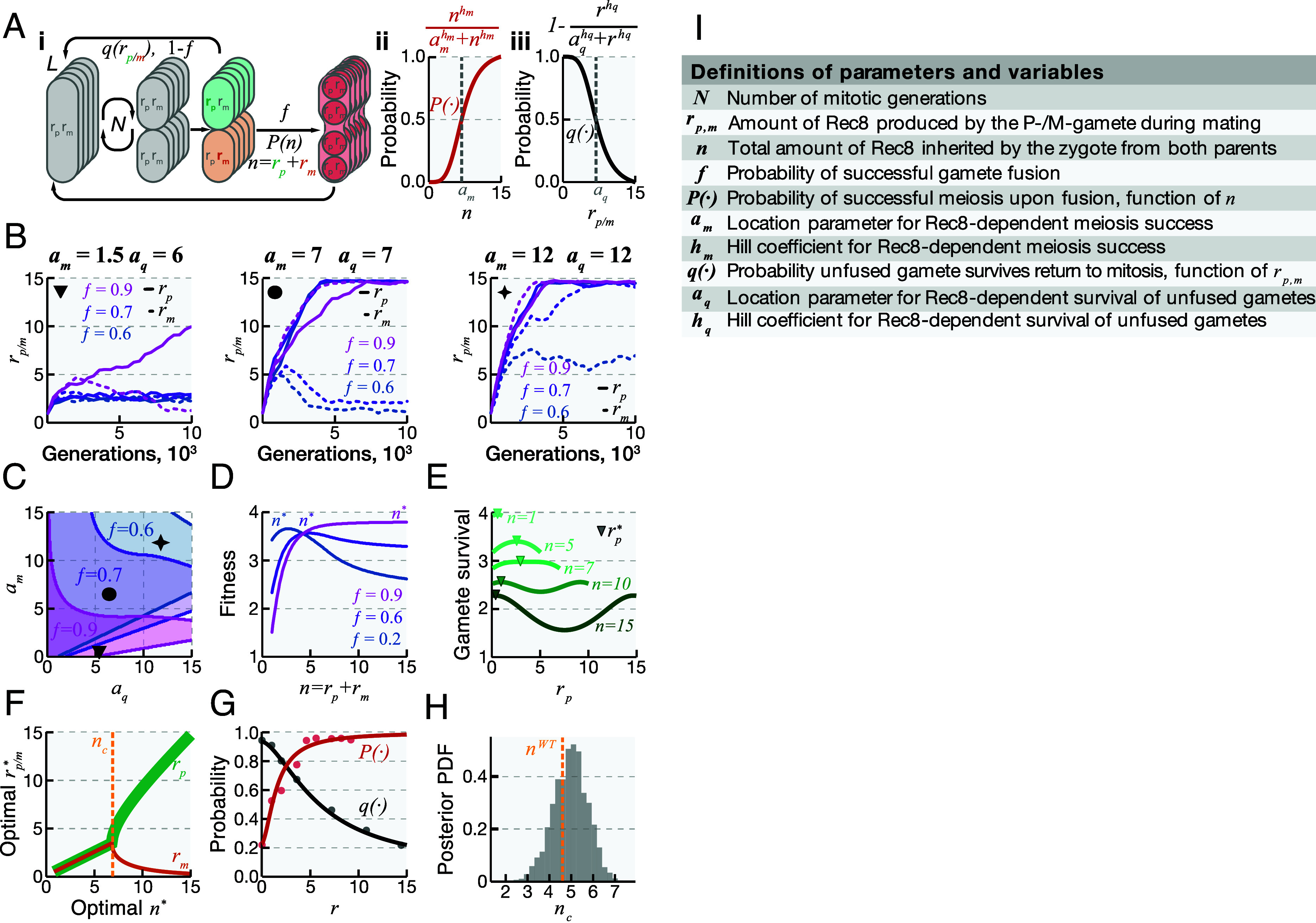
The selective pressures Rec8 places on gametes and zygotes drive asymmetric parental Rec8 contributions. (*A*) The schematic shows the lifecycle stages represented in the model (*i*). Each cell in a population of size *L* is associated with an evolving genotype $r_{p},r_{m}$ that defines the amount of the meiotic factor produced by the P- and M-parent during mating. Cells proliferate mitotically for *N* generations followed by sister cells mating step. We introduce a probability, f, that partners fuse during mating. If fusion is successful, zygotes receive ${n=r}_{p}+r_{m}$ meiotic factor investment and sporulate with probability $P\left(n\right)$, which we assume takes a sigmoidal form (*ii*, red line). Nonfused gametes return to mitosis with probability $q(r_{p/m})$, which we assume takes the sigmoidal form (*iii*, black line), and die otherwise. Parameter and variable definitions are given in panel *I*. (*B*) The average allocation of the meiotic factor in the P and M parents, $r_{p}$ and $r_{m}$ respectively, in populations evolving for 10,000 generations. The gamete that evolves the higher r value is designated as the P-parent ($r_{p}$, solid lines) and its partner as the M-parent ($r_{m},$ dashed lines). Depending on parameter choices the population evolves to a symmetric state where $r_{m}=r_{p}$ (when dashed and solid lines converge) or an asymmetric state where $r_{m}<r_{p}$ (dashed and solid lines diverge), suggesting that in theory asymmetry in meiotic factor contribution can theoretically be an evolutionary stable strategy under some conditions. The simulations were performed for three different fusion probabilities f (indicated by color) and for three different combinations of $a_{q}$ and $a_{m}$ parameters (indicated with ▼, ●, ✦ markers and values on the top) that define the shape of $P\left(\cdot \right)$ and $q\left(\cdot \right)$ functions. For simulation details, *SI Appendix*, *Supplemental Notes* *S2*. Parameter and variable definitions are given in panel *I*. (*C*) The regions in parameter space $a_{q}$ vs. $a_{m}$ that result in evolution of asymmetric parental investment as the optimal reproductive strategy (i.e., $r_{m}<r_{p}$; panel *B*). Here, the optimal genotype $\left(r_{p}^{*},r_{m}^{*}\right)$ was defined as asymmetric if $\frac{r_{p}^{*}}{r_{m}^{*}}>1.5$. Shaded regions correspond to those combinations of $a_{q}$ and $a_{m}$ which satisfy this condition, for given values of fusion probability f indicated by color. These quantifications suggest that the combined effects of f, $a_{q}$, and $a_{m}$ impact the likelihood for the evolution of asymmetric parental investment. Markers (▼, ●, ✦) indicate the parameter sets used in the evolutionary simulations in panel *B*. Parameter and variable definitions are given in panel *I*. (*D*) The graph shows the overall fitness measured as expected number of offspring defined by Eq. **1** in the main text, vs. total parental investment of meiotic factor n, for a given fusion efficiency f. We first determine the optimal genotype $\left(r_{p}^{*},r_{m}^{*}\right)$ given the requirement that $r_{p}^{*}+r_{m}^{*}=n$. These values are then used to compute the fitness as a function of n using $E\left(n,r_{p}^{*}\left(n\right)\right)=4fP\left(n\right)+2\left(1-f\right)\left[q\left(r_{p}^{*}\left(n\right)\right)+q\left(n-r_{p}^{*}\left(n\right)\right)\right]$, *SI Appendix*, *Supplemental Notes* *S2*. The plots suggest that as the probability of fusion increases the optimal total investment for zygotes increases. Parameter and variable definitions are given in panel *I*. (*E*) Gamete survival quantified as the expected number of offspring produced by unfused gametes, given by $2\left[q\left(r_{p}\right)+q\left(n-r_{p}\right)\right]$, vs. the P-parent investment $r_{p}$ for given values of total parental investment of meiotic factor for the zygote set at indicated values of $n=r_{p}+r_{m}$. The triangle lllzzzzl;Ksymmetric investment of the meiotic factor by the two parents is optimal. As n increases, the overall fitness of unfused gametes, $2\left[q\left(r_{p}^{*}\right)+q\left(n-r_{p}^{*}\right)\right]$, decreases and, importantly, the optimal $r_{p}^{*}$ bifurcates away from the equal parental contribution at the curve middle, indicating that an asymmetric investment of the meiotic factor by the two parents becomes optimal. Parameter and variable definitions are given in panel *I*. (*F*) The optimal meiotic factor contribution by each parent ($r_{p}^{*},r_{m}^{*}$) vs. the optimal total parental investment $n^{*}$. This is a summary plot of panel *E*, that allows visualization of the bifurcation point where gamete investment becomes asymmetric. Once the total investment is driven past a critical value $n_{c}$ (orange dashed line), asymmetric parental investment emerges as an optimal strategy (the green and orange lines branch off). Parameter and variable definitions are given in panel *I*. (*G*) The functional forms of the probability of zygote survival as a function of total Rec8 investment $P\left(\cdot \right)$ (red line) and of gamete survival as a function of the amount of Rec8 produced by gametes $q\left(\cdot \right)$ (black line) fitted to the experimental data (dots; *SI* *Appendix*, Fig. S7 *C*–*E*). Parameterization of the functions using experimental data is detailed in the *SI Appendix*, *Supplemental Notes* *S2*. Parameter and variable definitions are given in panel *I*. (*H*) The histogram shows the distribution of critical values $n_{c}$ derived for 10,000 parameter sets sampled from the inferred posterior distribution for the parameters of $q\left(\cdot \right)$ using experimental data. The critical $n_{c}$ value was defined to be the smallest n for which $\frac{r_{p}}{r_{m}}=1.5$. The dashed line indicates the total parental investment inferred for wild type matings ($n_{WT}=4.6A.U.$), which falls within the center of the predicted distribution suggesting that the costs and benefits of Rec8 expression identified experimentally are consistent with asymmetric gamete expression of Rec8. *SI Appendix*, *Supplemental Notes* *S2*. (*I*) The definitions of parameters and variables in the theoretical model.

Collectively, our experiments show that Rec8 expression prefusion produces opposing effects on the fitness of gametes and zygotes. This prompted us to hypothesize that, consistent with theoretical models for the evolution of anisogamy (4, 5, 37), such conflicting Rec8 roles drive the evolution of asymmetric Rec8 production between gametes. To rigorously test this idea—and because existing models are rooted in the life cycles of animal-like, multicellular organisms—we developed an evolutionary model based on the fission yeast life cycle (Fig. 4*A* and *SI Appendix*, *Supplemental Notes* *S2*). We consider a population where cells proliferate mitotically followed by a mating step. Each cell is associated with an evolvable genotype $\left(r_{p},r_{m}\right)$, which represents the quantity of Rec8 contributed at the point of fusion by the P- and M-gamete, respectively. Although our model is motivated, and subsequently parameterized, by the costs and benefits Rec8 elicits on gametes and zygotes, the framework we present is general and can be applied to the combined effect of meiotic factors beyond Rec8 and Rec11. We introduce probability f that partners fuse during mating. When fusion is successful, the cumulative amount of the Rec8 from both parents, ${n=r}_{p}+r_{m}$, specifies zygote fitness, which in our model defines the probability of successful meiosis, $P\left(n\right)$ (Fig. 4*A*). Alternatively, partners fail to fuse with probability 1-f and the amount of Rec8 in gametes, $r_{p/m}$, imposes a fitness cost. We model this cost with the probability that a gamete successfully returns to mitosis to produce daughter cells, $q(r_{p/m})$ (Fig. 4*A*). By subjecting the population to cycles of $r_{p},r_{m}$ mutation, asexual and sexual reproduction, we find that populations initially set to a symmetric state ($r_{p}=r_{m}$) can evolve to an asymmetric state ($r_{p}\neq r_{m}$; Fig. 4*B* and Movie S12). Thus, the fission yeast life cycle properties captured by our model allow evolution of asymmetric gamete investment.

Numerical simulations indicated that the evolution of asymmetric gametes depended on the probability f that partners fuse, and the Rec8-dependent fitness benefits and costs, specified by the $a_{m}$ and $a_{q}$ parameters that define the shape of the functions $P\left(\cdot \right)$ and $q\left(\cdot \right)$, respectively (Fig. 4*B*). We corroborated these results by noting that the expected number of offspring which originate from a genotype $\left(r_{p},r_{m}\right)$ after one life cycle, effectively defining its fitness, is given by (*SI Appendix*, *Supplemental Notes* *S2*)

[1] $$E(r_{p},r_{m})=2\left(1-f\right)[q\left(r_{p}\right)+q\left(r_{m}\right)]+4fP_{m}(r_{p}+r_{m}).$$

Using Eq. **1** we defined regions in the parameter space where gametes investment ratio, $\frac{r_{p}}{r_{m}}$, exceeded 1.5 (Fig. 4*C*, shaded areas), confirming that the fusion efficiency f and the parameters that determine the shape of $P\left(∙\right)$ and q(∙) collectively determine the optimal ratio between the two parental Rec8 contributions.

What sets the evolutionary optimum for the total amount of Rec8 provided by both parents? We evaluated fitness $E(r_{p},r_{m})$ in terms of the total parental investment, $n=r_{p}+r_{m}$, which provides benefits if partners fuse ($P\left(n\right)$ increases with n) and produces costs if fusion is unsuccessful ($2\left[q(r_{p})+q(n-r_{p})\right]$ decreases with increasing n). Balancing these opposing effects, the overall fitness $E(r_{p},r_{m})$ is maximized for a given value of n, which we term $n^{*}$ (Fig. 4*D*). The fusion efficiency f acts as a weighing factor between benefits and costs of Rec8 investment Eq. **1** and is thus crucial in determining the optimal total investment $n^{*}$ (Fig. 4*D*). For example, at low f, unfused gametes are numerous and the optimal total parental investment decreases because smaller n reduces the costs of returning to mitosis. As f increases, meiotic success of increasingly abundant zygotes dominates, and the overall fitness $E(r_{p},r_{m})$ is maximal for higher total investment n, increasing $n^{*}$ (Fig. 4*D*). In this way, the fusion efficiency f gates the contribution that the zygotic success $P\left(\cdot \right)$ has toward increasing the optimal total parental investment $n^{*}$ (Fig. 4*D*). Importantly, when selection drives the optimal investment $n^{*}$ above a critical value, $n_{c}$, the optimal gamete investments $r_{p/m}$ bifurcate and $r_{p}^{*}\neq r_{m}^{*}$ (Fig. 4 *E* and *F*). The bifurcation occurs when splitting the optimal total investment $n^{*}$ evenly is more costly than distributing it unevenly between unfused partners (Fig. 4*E*)—that is, when $2q(\frac{n}{2})<q(r_{p}^{*})+q(n-r_{p}^{*})$, as determined by the shape of the $q(r_{p/m})$ function and the value of n (*SI Appendix*, *Supplemental Notes* *S2*). Taken together, this analysis shows how the evolution of asymmetric Rec8 production in gametes critically depends on the fusion efficiency and quantitative effects of Rec8 on fitness of gametes and zygotes.

Are the Rec8 costs and benefits we identified experimentally sufficient to drive the evolution of asymmetric Rec8 provisioning? To address this question, we experimentally quantified P(n) and $q(r_{p/m})$. Specifically, we quantified the effect of Rec8 levels on the fitness of gametes and zygotes by measuring the spore viability and the unfused gamete survival for cells carrying different copy number of the *rec8* gene (*SI* *Appendix*, Fig. S7 *A*–*E*). We used Bayesian inference to parameterize P(·) and q(·) from this experimental data (Fig. 4*G* and *SI Appendix*, Fig. S7 *A*–*E* and *Supplemental Notes S2*). Using this parameterization, and the high fusion efficiency observed earlier (f=0.9; Fig. 3*A* and *SI* *Appendix*, Fig. S3*A*), we produced a posterior predictive distribution for the critical total parental investment $n_{c}$ above which we expect a substantial asymmetry in Rec8 investment to evolve (Fig. 4*H*). Crucially, the posterior predictive probability of $n_{c}$ being lower than the total Rec8 investment quantified from WT matings was 33.06% (Fig. 4*H*; $n_{\mathrm{WT}}=4.6\;a.u.$, *SI* *Appendix*, Fig. S7*A*), consistent with a high likelihood of asymmetry arising at the experimentally quantified Rec8 levels and fitness effects. Taken together, our theoretical model, experimental measurements, and statistical analysis show that an asymmetry in Rec8 investment is likely instigated by the conflicting selective pressures Rec8 places on gametes and zygotes.

## Discussion

Unlike asymmetrically expressed mating factors such as pheromone receptors, Rec8 expression is under opposing selective pressures in gametes and zygotes. Rec8 production prefusion, while indispensable for early zygotic development, imposes a cost on unfused gametes, creating a conflict that is minimized through asymmetric parental investment and specialization between closely related partners. The results of our experimentally parameterized model mirror predictions from the classic Parker-Baker-Smith model of anisogamy evolution (5, 37), and provide direct empirical evidence for one of their central assumptions: that asymmetries can emerge through trade-offs in gamete-level and zygote-level fitness. Interestingly, fission yeast parental asymmetries do not require complex differences in gamete size but molecular-level specialization toward zygote investment. Since mutating *mfm* genes alone alters Rec8 expression in gametes, limited changes in pheromone communication may promote parental asymmetry. For example, M-cell specific transcription factor Mc may increase M-pheromone production either directly (38, 39) or by downregulating general signaling factors (39, 40). Likewise, P-cell sensitivity to the M-pheromone might be reduced by the P-cell specific transcription factor Pc (41). Such limited regulatory asymmetries as a precursor to gamete dimorphism are consistent with mating-type loci in the anisogamous *Eudorina*, which contain only two sex-limited genes and two pairs of gametologs (i.e., genes with alleles in both mating haplotypes) (42). Functional complementation between transcription factors residing at mating-type loci of isogamous, anisogamous, and oogamous Volvocine species suggests that appearance of dimorphic gametes need not rely on their extensive sequence divergence but on changes in their regulatory networks (43). Asymmetry in Rec8 production through duplication of the fission yeast *mfm* genes would be consistent with this model. Extensive sex-specific allele differentiation may in fact arise after the dimorphic gametes appear, as proposed for the MAT3 gene of *Volvox carteri* (44).

Since gametic Rec8 production is regulated by the cascade that originates at the mating-type loci, selection for Rec8 expression dynamics might affect evolution of these loci (45), which have been proposed to be precursors of sex chromosomes (46–48) and to promote the evolution of anisogamy in isogamous ancestors (46, 49–51). Furthermore, the mating-type-biased selection pressures we observe might shape composition of populations, which could explain why P-cells may be underrepresented in the majority of fission yeast isolates (52) and the dominance of one mating type observed in medically relevant fungal species (53, 54). By studying Rec8 physiological roles across biological scales—from molecular and cellular functions to population dynamics—this work bridges cell biology and evolutionary theory, offering a mechanistic explanation for how functional differences between gametes may evolve before the appearance of morphological dimorphism.

## Materials and Methods

Yeast were cultured using standard media detailed Dataset S1. Fission yeast gene nomenclature, sequences, and annotations were obtained from the PomBase database (55). Strains used in the study are listed in the Dataset S2. Standard genetics manipulations were used to introduce and propagate genetic modifications, and all the genetic markers are detailed in Dataset S3. Experimental manipulations are detailed in the *SI Appendix*, *Supplemental Document* *S1*. Theoretical analyses are detailed in the *SI Appendix*, *Supplemental Notes* *S2*.

## Declaration of Generative AI and AI-Assisted Technologies

During the preparation of this manuscript, the authors used ChatGPT in order to improve text clarity and grammar. The authors subsequently reviewed and edited the content as needed and take full responsibility for the content of the manuscript.

## Supplementary Material

Appendix 01 (PDF)

Dataset S01 (XLSX)

Dataset S02 (XLSX)

Dataset S03 (XLSX)

Dataset S04 (XLSX)

Movie S1. **P-gametes induce expression of meiotic genes before partner M-gametes. (A)** The timelapse shows endogenous Rec8 tagged with sfGFP (green) during mating of homothallic wild-type cells. The M-gamete-specific *p^mam1^* promoter drives expression of cytosolic mTagBFP2 (blue), which we used to determine the gametes mating type (P, M) and time of fusion, which is observed as cytosolic mixing between partners. Brightfield micrographs are also shown (gray). The yellow dashed lines indicate a mating pair of interest. Note that cells show the faint background fluorescence in the green channel in the form of dots and strings, most likely corresponding to mitochondria. Note that Rec8-sfGFP produces a diffuse nuclear signal in addition to prominent nuclear foci that are consistent with reported Rec8 enrichment at centromeres^9^. Note the Rec8-sfGFP signal appearance prior to gamete fusion and first in the centrally positioned nucleus of the Pgametes, followed by M-gametes. Note the continued Rec8-sfGFP production in zygotes and that the protein is degraded during meiotic divisions and ahead of spore formation. **(B)** The timelapse shows endogenous Rep1 tagged with sfGFP (green) during mating of homothallic wild-type cells. The mTagBFP2 (blue) and the brightfield (gray) images, as well as yellow dashed lines, as in (A). Note that Rep1-sfGFP produces a diffuse nuclear signal that appears during mating and first in P-partners. Note that Rep1-sfGFP signal peaks shortly after partners fuse, followed by a signal decrease and complete degradation at meiotic divisions. **(C)** The timelapse shows endogenous Ste11 tagged with sfGFP (green) in homothallic wild-type cells immediately after the shift to nitrogen-free media and throughout sexual reproduction. Cells co-express mCherry (magenta) fused with the nuclear localization signal (NLS) that labels nuclei. The mTagBFP2 (blue) and the brightfield (gray) images, as well as yellow dashed lines, as in (A). Note that Ste11-sfGFP is already induced in cells that did not arrest the mitotic divisions, with comparable signal in the nucleus and the cytosol. Note that during mating, Ste11 levels and nuclear targeting increase, and then rapidly decrease upon gamete fusion as Ste11 is degraded in zygotes. The differences in Ste11 nucleocytoplasmic ratio in P- and M-gametes are reported in Fig. 1F, S2N. All timepoints are relative to the start of the timelapse. Scale bars are indicated.

Movie S2. **Pheromone communication regulates the bias in meiotic gene expression between P- and M-gametes. (A)** The timelapse shows endogenous Ste11 tagged with sfGFP (green) in homothallic *mfm1Δ mfm3Δ* mutants immediately after the shift to nitrogen-free media and throughout sexual reproduction. The M-gamete-specific *p^mam1^* promoter drives expression of cytosolic mTagBFP2 (blue), which we used to determine the gametes mating type (P, M) and the time of fusion, which is observed as cytosolic mixing between partners. Cells coexpress mCherry (magenta) fused with the nuclear localization signal (NLS) that labels nuclei. Brightfield micrographs are also shown (gray). The yellow dashed lines indicate a mating pair of interest. Note that Ste11-sfGFP is already induced in cells that did not arrest the mitotic divisions, with comparable signal in the nucleus and the cytosol. The differences in Ste11 nucleocytoplasmic ratio in P- and M-gametes are reported in Fig. S3B-S3C.**(B)** The timelapse shows endogenous Rep1 tagged with sfGFP (green) during mating of homothallic *mfm1Δ mfm3Δ* mutants. The mTagBFP2 (blue) and the brightfield (gray) images, as well as yellow dashed lines, as in (A). Note that Rep1-sfGFP produces a diffuse nuclear signal that appears during mating and first in M-partners. **(C)** The timelapse shows endogenous Rec8 tagged with sfGFP (green) during mating of homothallic *mfm1Δ mfm3Δ* mutants. The mTagBFP2 (blue) and the brightfield (gray) images, as well as yellow dashed lines, as in (A). Note the Rec8-sfGFP signal appearance prior to partner fusion and first in M-gametes. All timepoints are relative to the start of the timelapse. Scale bars are indicated.

Movie S3. **Rec8 is required for accurate meiotic segregation of chromatin**. Timelapses show localization of the Hht1 histone H3^6^ fused to the red fluorescent protein RFP (magenta) during mating of either wild-type or *rec8Δ* mutants, as indicated. Brightfield channel is shown (gray). The yellow dashed lines indicate a mating pair of interest. Note that zygotes with two wild-type parents (top row), or mutant *rec8Δ* M-parent (second row), produce four equal chromatin masses of Hht1-RFP (arrowheads), whereas meiotic divisions in zygotes with *rec8Δ* P-parent (third row), or two *rec8Δ* parents (bottom row), segregate Hht1-RFP into aberrant number of uneven chromatin masses (arrowheads). Also note that after cells fuse, partner nuclei undergo karyogamy in both wild-type and mutant zygotes. All timepoints are relative to the start of the timelapse. Scale bars are indicated.

Movie S4. **Rec8 expression in gametes promotes accurate sporulation**. Timelapses show Rec8-sfGFP (green) expressed from the M-gamete-specific *p^mam1^* (**A**), mating-induced *p^pak2^* (**B**), and zygotic *p^mei3^* (**C**) promoter during mating between parents that lack the native *rec8* gene. The cells co-express the mTagBFP from either the P-cellspecific *p^map3^* (**A, B**) or the M-gamete-specific *p^mam1^* (**C**) promoter, which we used to determine gametes mating type (P,M) and the time of cell fusion. Brightfield micrographs are also shown (gray). The yellow dashed lines indicate a mating pair of interest. Note that Rec8-sfGFP is induced from the *p^mam1^* promoter hours before cell fusion and in Mgametes only, from the *p^pak2^* promoter in both gametes during mating, and from the *p^mei3^* promoter at the time of fusion. Note that four spores (white arrowheads) form in zygotes driving expression of Rec8-sfGFP from *p^mam1^* or *p^pak2^* promoters, whereas the zygote driving expression of Rec8-sfGFP from *p^mei3^* promoter produces aberrant number of spores. All timepoints are relative to the start of the timelapse. Scale bars are indicated.

Movie S5. **P- and M-alleles produce distinct amounts of Rec8 at the onset of premeiotic S-phase**. Timelapses show Rec8-sfGFP (green) expressed from either one or both parental alleles, as indicated. Cells co-express mCherry-Pcn1 (magenta), which during DNA-replication forms foci (magenta arrowheads) and a uniform nuclear signal outside S-phase. The Mgamete-specific *p^mam1^* promoter drives expression of mTagBFP2 (blue), which we used to determine gametes mating type (P,M) and time of cell fusion. Brightfield micrographs are also shown (gray). The yellow dashed lines indicate a mating pair of interest. Note that the Rec8-sfGFP signal at the onset of S-phase (green arrowheads) is lower when produced by the M-allele alone (bottom) than that produced by the P-allele alone (middle) or both parental alleles (top). All timepoints are relative to the start of the timelapse. Scalebars are indicated.

Movie S6. **Delaying the pre-meiotic S-phase allows for increased Rec8 production from the M-allele ahead of DNA replication**. Timelapses show Rec8-sfGFP (green) expressed from the M-allele in indicated crosses where P-gametes lack the *rec8* gene, and the *mei3* gene is either intact or deleted. Cells co-express mCherry-Pcn1 (magenta), which during DNA-replication forms foci (magenta arrowheads) and a uniform nuclear signal outside S-phase. The M-gamete-specific *p^mam1^* promoter drives expression of mTagBFP2 (blue), which we used to determine gametes mating type (P,M) and time of cell fusion. In the brightfield channel (gray), the yellow lines indicate a mating pair of interest. Note that deleting the *mei3* in P-gametes delays premeiotic S-phase, which allows for increased Rec8-sfGFP production (green arrowheads) from the M-allele by the time DNA replication starts. Note the defective spore number (white arrowheads) in crosses where P-gametes lack the *rec8* gene only, and formation of four spores in zygotes that lack *rec8* and *mei3* genes. All timepoints are relative to the start of the timelapse. Scale bars are indicated.

Movie S7. **Native Rec8 levels are required for tight cohesion at chromosome arms**. The timelapses show mating between indicated wild-type, *rec8Δ and rec11Δ* partners where P-gametes carry the *lys3* locus labeled with the *parS^c3^*/ParB^c3^-eGFP (green) and M-gametes carry the lys3 locus labeled with the *parS^c2^/ParB^c2^*-mCherry-NLS (magenta). Brightfield micrographs are also shown (gray). Note that the *lys3* locus-recruited *parS^c3^*/ParB^c3^-eGFP (arrowheads) produces a single dot prior to meiosis in wild-type zygotes but two dots in zygotes that lack either parental *rec8* allele. All timepoints are relative to the start of the timelapse. Scale bars are indicated.

Movie S8. **Zygotes lacking parental rec8 alleles undergo aberrant, equational meiosis I**. The timelapses show mating between indicated wild-type, *rec8Δ and rec11Δ* partners where P-gametes carry the *rec6* locus labeled with the *parS^c3^*/ParB^c3^-eGFP (green) and M-gametes carry the rec6 locus labeled with the *parS^c2^/ParB^c2^*-mCherry-NLS (magenta). Brightfield micrographs are also shown (gray). Note that the rec6 locus-recruited *parS^c3^*/ParB^c3^-eGFP (arrowheads) produces a single dot prior to meiosis in wild-type zygotes but two dots in zygotes that lack either parental rec8 allele. Also note that at meiosis I, in wild-type zygotes the rec6 loci co-segregate and in *rec8* mutants the foci separate. All timepoints are relative to the start of the timelapse. Scale bars are indicated.

Movie S9. **Zygotes lacking Rec11 fail to segregate homologues**. The timelapses show mating between wild-type or *rec11Δ* partners where both gametes carry the rec6 locus labeled with the *parS^c3^*/ParB^c3^-eGFP (green). Brightfield micrographs are also shown (gray). Note that during meiosis I, in wild-type zygotes the pairs of *rec6* loci segregate to opposite poles (homologue disjunction) and that in *rec8* mutants the four *rec6* loci co-segregate together (homologue non-disjunction).

Movie S10. **Mating produces a subpopulation of P-gametes that induce Rec8 expression but fail to fuse with a partner**. The timelapse shows endogenous Rec8 tagged with sfGFP (green) during mating of homothallic wild-type cells. The M-gamete-specific *p^mam1^* promoter drives expression of cytosolic mTagBFP2 (blue), which we used to determine the gametes mating type (P, M) and time of fusion, which is observed as cytosolic mixing between partners. Brightfield micrographs are also shown (gray). The yellow dashed lines indicate the three cells that engage in mating. Note that two P-gametes induce Rec8-sfGFP expression during competition for the same M-partner. All timepoints are relative to the start of the timelapse. Scale bars are indicated.

Movie S11. **Rec8 expression during mating causes mitotic and growth defects in gametes that return to proliferation**. The timelapse shows homothallic cells carrying endogenous *rec8* tagged with sfGFP. Cells were induced to mate but the mating was interrupted by returning cells to growth conditions at the start of the imaging. We show the Rec8-sfGFP (green) upon return to growth media (first timepoint) and brightfield images (gray) for all timepoints. Note that Rec8-sfGFP is expressed in several unfused gametes. Note that the majority of unfused gametes resume growth and mitotic divisions. In the left panel, note that the outlined gamete expresses Rec8-sfGFP and that it lyses at first division. In the right panel, note that the outlined gamete expresses Rec8-sfGFP and that one of its daughter cells arrests growth. All timepoints are relative to the start of the timelapse. Scale bar is indicated.

Movie S12. **Evolution of asymmetric parental investment strategy using theoretical model of the fission yeast lifecycle**. Each magenta dot represents an individual genotype (*r_p_, r_m_*) evolving in the 2- dimensional genotype space according to our theoretical model. We use the colorscale to associates points in the genotype space with the expected number of offspring given by *E(r_p_, r_m_*) = 2(1 − *f*)[*q* (*r_p_*) + *q* (*r_m_*)] + 4*fp_m_* (*r_p_* + *r_m_*), and hence visualize the “fitness landscape”. The population is initialized with 10,000 individuals with an identical, symmetric genotype (1,1). Each frame represents a step of 100 evolutionary generations.

## Data Availability

The code related to the theoretical analyses is available at the GitHub repository https://github.com/harrybooth/Fission-Yeast-Rec8 (56). Custom scripts are compiled into a GUI-based tool, VLabApp, available at https://github.com/vjesticalab/VLabApp (57). Sequencing results are available under GEO accession identifier GSE293837 (58). All plasmids and fission yeast strains are available upon reasonable request. All data is available upon reasonable request. All other data are included in the manuscript and/or supporting information.
